# Supplementary material for: Hematological and coagulation alterations and splenic response following Macrovipera lebetina obtusa envenomation: Evaluation of ovine-derived experimental antivenom
Source: PLoS Negl Trop Dis. 2025 Nov 11;19(11):e0013724. doi: 10.1371/journal.pntd.0013724 (PMC12626325; doi:10.1371/journal.pntd.0013724)
Supplement: Attachment — Submitted filename: Response to Reviewers.docx [file pntd.0013724.s004.docx]

Response to Reviewers

Honorable sir,

Thank you very much for very detailed analysis of our work: indeed, all remarks of the reviewers are very relevant and constructive. Now in this annotated letter let us describe point-by-point any corrections, which we’ve done according the issues raised in your comments, addressing each point individually.

**Methods:**

**Reviewer N1**

1. Could the ambient temperature during vacuum drying of the venom have affected the enzymatic activity?

The vacuum-drying process was conducted under controlled laboratory conditions at room temperature (21–23°C), which is consistent with standard venom preservation protocols. Furthermore, the vacuum-dried venom’s enzymatic activity was tested earlier (Kishmiryan, A., et al. J Venom Res, 2021, Vol 11, 7-15. PMID: 33747434), as well as the overall LD50 versus lyophilized venom.

2. Considering that antivenom administration can mitigate some systemic effects, but not the local effect, what is the rationale for intradermal injection of antivenom 10 minutes after venom injection?

The intradermal administration of antivenom 10 minutes post-envenomation was performed to simulate a clinically relevant “rescue” treatment (or so-called “challenge then treat” experimental mode) rather than preincubation, reflecting realistic therapeutic intervention conditions. This timing allows local venom diffusion and tissue interaction to occur before treatment, enabling evaluation of the antivenom’s effectiveness in limiting local damage once envenomation has already started. The approach also aligns with earlier experimental and clinical observations suggesting that local antivenom delivery may enhance neutralization of tissue-bound toxins and reduce dermal necrosis when combined with systemic administration. As mentioned in the Discussion, antivenom administration in Middle Asia in the 70s and 80s of the 20th century was initially administered locally ASAP in the field, then intravenously in the hospital. Concerning the statement “that antivenom administration can mitigate some systemic effects, but not the local effect”, unfortunately, we could not find any published evidence in the Western scientific press. So, it is important to check empirically and investigate further in more detail.

**Reviewer N2**

1. Hypothesis Statement.

While the objectives are clearly articulated and logically derived from the contextual background, the manuscript does not explicitly present a formal, testable hypothesis within the Introduction. Articulating such a hypothesis would strengthen the scientific rigor and focus of the study.

Thank you for this valuable remark, the corresponding corrections were added to the Introduction.

2. Study Design

The study design is comprehensive and methodologically robust, aligning well with the stated objectives of characterizing the effects of *Macrovipera lebetina obtusa* venom and assessing antivenom efficacy. The chosen methodologies are appropriate for addressing the research questions posed.

Thank you for articulating the appropriateness of the study design.

3. Study Population
   The study population is clearly described and well-suited to the research aims. The selection criteria and characteristics of the population are appropriate for investigating the effects of envenomation and antivenom intervention in the context of neglected tropical diseases.

We are grateful for your assessment.

4. Sample Size and Statistical Power
   However, the reported sample size appears insufficient to ensure adequate statistical power for robust hypothesis testing, particularly given the variability inherent in toxinology and neglected tropical disease research. Increasing the sample size would enhance the reliability and generalizability of the findings.

The experimental design was based on 3R-refined WHO-recommended protocols, where the optimal group size for antivenom testing was accepted as 5-6 animals (Guidelines for the production, control and regulation of snake antivenom immunoglobulins). In: WHO Expert Committee on Biological Standardization: sixty-seventh report. Geneva: World Health Organization; 2017: Annex 5, pages 317, 318 (WHO Technical Report Series, No. 1004; <https://apps.who.int/iris/handle/10665/255657> , accessed 13 June 2022)).

5. Statistical Analysis

The statistical analyses employed are appropriate and correctly applied to the research questions and experimental design. However, the authors did not explicitly address the assumptions underlying the use of ANOVA. A discussion of these assumptions and how they were verified would improve the transparency and validity of the statistical approach.

The appropriate additions (see Figure and Table legends) were done.

6. Ethical and Regulatory Compliance

The study appears to adhere to all major ethical and regulatory requirements, with evidence of rigorous oversight and compliance with international standards for research involving potentially hazardous biological materials.

Thank you for your assessment.

**Results:**

**Reviewer N1**

1. Would be valuable if the authors add to Table 1 the measurements obtained from different timepoint (i.e., 1st day, 3rd day, and 7th day).

The experimental approach for these series implemented via cardiac puncture which is terminal for the animals, so following the 3R-refined protocols we have done the analysis of first day timepoint and only two animals for the third day to be sure that blood parameters returned to the standard frames in this period, while after these normal results seventh day timepoint analysis seems to us the unnecessary euthanizing of animals.

2. The authors state that " One clotting measure, the prothrombin time, stayed abnormal even after antivenom treatment, showing that the venom’s effects were only partly neutralized." The neutralization limitation of different antivenom formulations (complete IgG, F(ab’)2, etc.) is a common question in toxinology. I recommend that the authors discuss the secondary effect of SVMP and SVSP on hemostatic parameters. Since SVSP activates the coagulation cascade, antivenom is expected to have no effect on hemostatic parameters directedly dependent on the toxin’s activity.

The mentioned remarks were added to the Discussion.

**Reviewer N2**

1. Alignment of Analyses with Methodology
   The analyses presented in the manuscript are consistent with the analysis plan outlined in the methodology section. However, a minor limitation is the absence of explicit identification of the statistical tests employed in the results section. Clearly naming these tests would enhance transparency and reproducibility.

We added this information to the figures and table’s legends.

2. Addressing Outcomes and Contextualization
   All primary outcomes described in the methods are thoroughly addressed in the results, and the findings are effectively contextualized within the broader objectives of the study. This approach strengthens the manuscript’s contribution to the field and situates the results within the current landscape of Neglected Tropical Diseases research.

Thank you for your positive assessment.

3. Quality of Tables and Figures
   The tables and figures included in the manuscript are of high quality and clarity, meeting the standards expected in leading journals focused on Neglected Tropical Diseases. Their presentation facilitates comprehension and supports the effective communication of key findings.

Thank you for your positive assessment.

**Conclusions:**

**Reviewer N1**

1. The authors suggest that local injection of antivenom would have clinical value. In clinical settings, the patient arrives hours after the bite. How could an intervention 10 minutes after venom challenge have translational potential?

The local injection is discussed only for the local-damage neutralization hypothesis, whereas systemic pathological symptoms are neutralized during i.v. administration in the clinics. Moreover, here we are discussing blood flow in mice or rats, for which, given the average circulation time and much higher metabolic rate of small rodents, a 10-minute delay may be comparable to a spreading effect in the human organism, where it will take a few hours.

**Reviewer N2**

1. Strength of Conclusions

The study’s conclusions are clearly and directly supported by the data presented. The evidence for hemoconcentration, coagulopathy, and persistent tissue damage is robust and well-documented. The nuanced interpretation of the partial efficacy of antivenom, along with the call for adjunctive therapies, is well justified by the results and aligns with current scientific understanding in the fields of Neglected Tropical Diseases and viper envenomation.

Thank you for positive assessment.

2. Discussion of Limitations
The authors have appropriately acknowledged the biological and therapeutic limitations of antivenom treatment, as well as the complexity of venom pathophysiology. However, the manuscript would benefit from a more explicit and structured discussion of analytical and methodological limitations, including sample size, statistical assumptions, and the inherent constraints of animal models. Addressing these aspects would further strengthen the transparency and rigor of the study.

Thank you for the positive assessment. The Discussion section is structured primarily for data analysis, and with some requested additions, it could become too long if we discuss these aspects here. Instead, we’ve decided to add a substantial portion of the overview of existing antivenom limitations to the Introduction, which was quite generally restructured and edited.

4. Advancement of Knowledge

The discussion clearly articulates how the data advance our understanding of viper envenomation and its management. The authors thoughtfully situate their findings within the broader context of Neglected Tropical Diseases, highlighting the implications for both research and clinical practice.

Thank you for positive assessment.

5. Translational Relevance

The manuscript effectively bridges the gap between bench research and its implications for health systems, clinical practice, and policy in the context of Neglected Tropical Diseases. This translational perspective is particularly valuable given the ongoing challenges in addressing these diseases in resource-limited settings.

Thank you for positive assessment.

**Editorial and Data Presentation Modifications?**

**Reviewer #1:** 
Use *Macrovipera lebetina obtusa* on the first use and then abbreviate as M. l. obtusa or M. lebetina. Avoid the MLO nomenclature as it is not a good practice.

All changes have been performed throughout the manuscript. However, since the majority of the figures are embedded in the text mentioned MLO, we have to preserve this abbreviation in the figure legends only.
In line 33, correct the “it unable” to “it was unable”. Done.
In line 98, close the parenthesis in anticoagulation. Done.
In line 115, define CBC at first use. Afterward use the abbreviation instead of repeating. Done.
In line 125, use italic for *Macrovipera lebetina obtusa*. Done.
In line 151, add a space in the between the number and the unit “(24 hours)” for consistence. Done.
In line 189 use “euthanized” instead of “sacrificed”. Done.
The Figure 4 need the A and B marks in the panels. Additionally, the legend needs a correction In line 356. Check the following sentence: “antivenom injection with 10 10-minute delay.”

The marks have been added, and the repetition in the legends has been removed as suggested.
Legends of Figures 3 and 5 are confusing. The legends describe the magnification for upper panel as 400x and for the lower panel as 100x, but the image seems to be the opposite.

Thank you for highlighting these typos. The corrections have been made both for scale bars and magnifications in figures 3 and 5.
Place the Table 2 at the results where it is first mentioned. Done.

**Reviewer #2:** In addition to the comments I have included in this form, I have uploaded an annotated version of the article.

Comment 1. How many (snakes)? Male? Female? Both? Age? Approximately 25 animals of both sexes and middle-aged snakes (~4-6 years) were used for venom milking.

Comment 2. Create a subsection titled, e.g., "2.3 experimental antivenom"to introduce more details about antivenom as seen in: Avagyan et al., Histopathological changes of mice tissues in course of the envenomation by the Macrovipera lebetina obtusa venom and the neutralizing effect of the ovine-derived experimental antivenom. Toxicon 247 (2024) 107821. Although the respected reviewer referred to our recent article, where there actually is no subsection “Experimental antivenom” in the Methods, we’ve added a subsection here to briefly describe the methodology by which the ovine-derived experimental antivenom used in this investigation was developed. More in detail, it was described in Kishmiryan A, et al. J Venom Res. 2021;11:7-15 (reference included in the list).

Comment 3. What is paraplast? Paraplast is a refined mixture of highly purified paraffin-containing plastic polymers used for tissue sample embedding.

Comment 4. Reference. The requested reference has been added (see line 207, N30 in the list).

Comment 5. Reference. The requested reference has been added (see line 207, N30 in the list).

Comment 6. From where, veninpucture? Cardiac puncture (terminal), under anesthesia. The phrase is added to the method.

Comment 7. References. The requested reference has been added (see line 219, N31 in the list), and the methodologies have been used with minor modifications.

Comment 8. *Lebetina, exerted, examined* and other changes. The typos have been corrected.

Comment 9. Expand the discussion of coagulopathy induced by *Viperidae* venoms. Done (see line 412).

Comment 10. References. The appropriate references have been added (see lines 425, 429, 433, 439, 460, 468, 477, 482).

**Our sincere gratitude to the Reviewers for their detailed analysis of the work and deep interest in the scientific problems and approaches raised in this manuscript. The certain remarks we’ll accept as a proposition to continue this investigation with the post-injection scheme of experiment to assess the pharmacokinetics of the antivenom in the organism of prey.**
